# Multi-omics Analysis of Microenvironment Characteristics and Immune Escape Mechanisms of Hepatocellular Carcinoma

**DOI:** 10.3389/fonc.2019.01019

**Published:** 2019-10-15

**Authors:** Wenli Li, Huimei Wang, Zhanzhong Ma, Jian Zhang, Wen Ou-yang, Yan Qi, Jun Liu

**Affiliations:** ^1^Department of Reproductive Medicine Center, Yue Bei People's Hospital, Shaoguan, China; ^2^Morning Star Academic Cooperation, Shanghai, China; ^3^State Key Laboratory of Medical Neurobiology, Department of Integrative Medicine and Neurobiology, School of Basic Medical Sciences, Institute of Brain Science, Shanghai Medical College, Fudan University, Shanghai, China; ^4^Department of Clinical Laboratory, Yue Bei People's Hospital, Shaoguan, China; ^5^The Second Clinical Medical College, Zhujiang Hospital, Southern Medical University, Guangzhou, China; ^6^Yunnan Provincial Key Laboratory of Traditional Chinese Medicine Clinical Research, First Affiliated Hospital of Yunnan University of Traditional Chinese Medicine, Kunming, China

**Keywords:** hepatocellular carcinoma, molecular subtype, bioinformatics analysis, cancer stage, immune escape, immune-enhanced subtype

## Abstract

The immune environment in primary tumor has a profound impact on immunotherapy. However, the clinical relevance of immune environment in hepatocellular carcinoma (HCC) is largely unknown. Here, the immune profile and its clinical response in HCC were investigated. The gene expression profiles of 569 HCCs from three cohorts (The Cancer Genome Atlas, TCGA, *n* = 257; Gene Expression Omnibus, GEO, *n* = 170; International Cancer Genome Consortium, ICGC, *n* = 142) were used in the current study. Five gene expression subtypes (C1–C5) responsible for global immune genes were identified in HCCs at stage I/II. It was found that subtype C4 was associated with upregulation and subtype C5 was associated with downregulation of immune profiles in most metagenes. Immune-correlation analysis of the five subtypes demonstrated that C3 and C4 had higher immune score and better prognostic outcome, as compared with other subtypes. Moreover, the mutation frequencies of TP53, CTNNB1, and AXIN1 had significant difference in the five subgroups. Further, the expression of PDCD1, CD274, PDCD1LG2, CTLA4, CD86, and CD80 was higher in subtype C4 in comparison with the other subtypes. The WGCNA of immune-related genes in the five subtypes revealed that blue and turquoise modules were positively correlated with subtype C4 and were associated with 12 common pathways in the KEGG database. These results were validated in external cohorts from the NCI (National Cancer Institute) cohort (GSE14520) and the ICGC (International Cancer Genome Consortium) cohort. In summary, one immune-enhanced subtype and one immune-decreased subtype having different immune and clinical characteristics may provide guidance for developing novel treatment strategies for immune system malfunction-related cancer.

## Introduction

Hepatocellular carcinoma (HCC) is ranked as the second cause of cancer-related mortality and the fifth most common malignancy in patients worldwide (1). It was estimated in 2012 that around 700,000 people die of HCC every year worldwide (2). The incidence of HCC varies with geographical area, sex, age, and risk factor related to cancer development (3, 4). Despite advances in HCC treatment, such as liver transplantation, surgical resection, and radiofrequency ablation, the prognosis of HCC patients is still low, with a 5-year survival rate of <30% (5). Moreover, the tumor heterogeneity and microenvironment cells also play a critical role in treatment or analysis of tumor stage in HCC (6). Notably, the molecular mechanisms by which the microenvironment cells regulate the development of HCC have not been extensively explored.

HCCs are mostly caused by chronically inflamed liver and are considered as typical immunogenic cancers (7). Immune procedures play important roles in HCC carcinogenesis and progression. Immune suppressor cells, including regulatory T cells (Tregs), tumor-associated macrophages (TAMs), and myeloid-derived suppressive cells (MDSCs) in the HCC tumor microenvironment (8), could result in tumor immune evasion or immune escape by interfering with immune surveillance (9). Moreover, the immune checkpoint, which is known to modulate different stages and signaling procedures of the immune response, is one of the mechanisms of escaping anti-cancer immune surveillance. It has been revealed that immune treatment targeting coinhibitory receptors (i.e., CTLA4 and PD1) increase immune response by inhibiting the immunosuppressive mechanisms in several cancers, such as metastatic melanoma and lung cancer (10, 11), but immunotherapy has not been successfully explored in HCC for decades (12). In addition, the expression profile and clinical relevance of immune checkpoint molecules in HCC have not been studied extensively.

Previous studies explored the effects of tumor microenvironment in HCC. Survival outcome of HCC patients was investigated using immunohistochemistry and quantitative PCR techniques to elucidate the underlying immune gene expression profiles (13). The expression of 49 immune genes was detected in 68 HCC patients. However, the sample size may have reduced the reliability of the conclusions. Moreover, qPCR method detected only two tumor phenotypes (proliferation and apoptosis) and 49 immune genes that were associated with the survival outcome of HCC patients. There is a need to comprehensively detect all the tumor phenotype and global immune profiles using high-throughput techniques. Transcriptome analysis using next-generation sequencing and microarray profiling is a powerful method for systemically exploring the tumor microenvironment. Previous studies revealed the presence of microenvironment cells in tumor tissues via RNA sequencing and microarray analysis of their underlying expression profiles (14, 15). Using microarray analysis and qRT-PCR technique, a unique inflammation/immune response-associated signature of the liver microenvironment was found to be a predictor of venous metastases, recurrence, and prognosis of HCC (16). However, due to the cross-validation in training set and lack of external validation, a larger HCC cohort is needed to further validate the conclusions.

The current research aimed to comprehensively explore the heterogeneous immune microenvironment phenotypes and their associated clinical relevance in HCC at stage I/II. The Cancer Genome Atlas (TCGA) cohort was used to successfully classify 257 HCC samples into five consensus molecular subtypes of tumors with potential immune escape mechanisms and genomic drivers underlying the gene expression profiles of global immune genes. Both immune-enhanced and immune-decreased subtypes were identified in HCC. Moreover, the five subtypes were validated using an external dataset from the NCI (National Cancer Institute) cohort and the ICGC cohort.

## Methods and Materials

### Genomic Analysis of Immune Genes

Thirteen immune metagenes were obtained from an immune gene set in the TIMER database (17, 18) to reflect the types and functions of various immune genes. Median level of gene expression reflected the scores of the metagenes. In addition, scores of 10 types of immune-related cells were calculated using the MCP counter R package (19).

### HCC Sample Datasets

The RNA-seq data, SNP data, clinical data, and immune-associated genes in HCC were retrospectively collected from the TCGA database (20) (https://cancergenome.nih.gov/) and the NCBI GEO database (21) (https://www.ncbi.nlm.nih.gov/geoprofiles/). The gene expression profiles of clinical data from 257 HCCs were retrieved from the TCGA database with the following criteria: (a) at stage of I and II; (b) accompanied by detailed follow-up information; (c) accompanied by HCC gene expression profiles; (d) genes with expression levels >0 in each sample accounting for more than 30% of the genes identified in the immune gene set (22, 23). Disease-free survival (DFS) and progression-free survival (PFS) were analyzed for the TCGA cohort.

The external validation cohort included 445 HCCs that were collected by the NCI from the GSE14520 dataset (24, 25). A total of 170 HCCs with the desired gene expression profiles and at stage I and II were included in the analysis. The other external validation cohort was from ICGC, which included 142 HCCs (Table S1).

### Data Preprocessing

The RNA-seq data were analyzed using the Illumina platform. The fragments per kilobase of gene per million fragments mapped with upper quartile normalization (FPKM-uq) and single-nucleotide polymorphism (SNP) from TCGA Data Portal were downloaded. Next, gene annotation was performed using the Ensemble database. The gene expression value was log2-transformed for further exploration. The gene expression of the NCI cohort was calculated using Affymetrix HT Human Genome U133A Array and Affymetrix Human Genome U133A 2.0 Array. The expression data and related clinical data of the validation set were obtained from the Gene Expression Ominibus (GEO) (GSE14520). Probe annotations were downloaded from the GEO database. The mRNA expression data and clinical information were downloaded from ICGC. Entrez Gene IDs were used for gene expression data analysis in the three cohorts. The corresponding scores of six types of immune-related HCC cells in each sample were downloaded from Timer database (https://cistrome.shinyapps.io/timer/). The immune score, the matrix scores, and identification of immune gene expression profiles were achieved using the R package estimate (19).

### Identification of HCC Subtypes Based on the Immune Genes

The ConsensusClusterPlus package (26) was utilized to perform consistent clustering and screening of molecular subtypes based on immune gene expression profiles. The Euclidean distance is used to calculate the similarity distance between samples (27), and K-means is used for clustering (28). The clustering was performed using 100 iterations, with each iteration containing 80% of samples. The optimal cluster number was determined by cumulative distribution function (CDF) curves of the consensus score (29). SigClust analysis was applied for pairwise comparisons to test the significance of clustering among identified subtypes (30). The genes with high expression in some subtypes were identified using Kolmogorov–Smirnov test. Bonferroni correction was applied for multiple testing. The Benjamini–Hochberg method was used to calculate the false discovery rate (FDR), and genes with FDR <0.05 were considered to be significantly upregulated genes. The top 100 upregulated genes in each subtype were selected and subjected to three-dimensional principal component analysis (PCA) to distinguish different molecular subtypes (31). PCA is a statistical method used to determine the main variables in a multidimensional dataset, which represent the differences among observations (32). Thus, several key clusters rather than all of the selected upregulated genes were utilized to classify the subtypes using PCA.

### Gene Co-expression Network Analysis

The common pathways related to the six gene modules were determined using the WGCNA R package (33). The construction of the WGCNA network and module detection were conducted using an unsigned type of topological overlap matrix (TOM), a power β of 3, a minimal module size of 30, and a branch merge cutoff height of 0.25. KEGG enrichment analysis was performed using R package clusterProfiler with FDR < 0.05. The most significant correlated genes with WGCNA edge weight >0.15 were visualized using Cytoscape 3.7.1 (34).

### Validation of Five Immune-Related Subtypes

To validate five immune-related subtypes identified from the TCGA cohort, the genes in the co-expression gene modules (blue, brown) that are closely related to the C3 and C4 subtypes were selected, and the correlation between the genes and the modules was calculated, and then the cancer samples in NCI cohort were classified based on the featured genes with the correlation coefficient >0.8. Moreover, the gene expression profiles extracted from the validation set were used to classify the samples by the Support Vector Machine (SVM). To further validate the five immune-related subtypes, the normalized data of 445 samples were downloaded from the GSE14520 dataset and 142 samples from the ICGC. A total of 312 samples at stages I and II and containing the gene expression profiles of the featured genes were extracted and classified using SVM.

### Statistical Analysis

The relationship between clinical variables and subtypes was analyzed by chi-square test or Fisher's exact test. Multiple testing was corrected by Benjamini–Hochberg's FDR. Kaplan–Meier curves and log-rank test were used to compare the 10-year DFS and PFS rates of the five immune subtypes. All tests were two sided, and *P* < 0.05 was considered to be statistically significant. Student's *t*-test was used to compare the immune scores and expression values of checkpoint genes among the HCC subtypes. The FDR correction was performed to decrease false-positive rates in multiple tests. All statistical tests were two-sided. All statistical analyses were performed using R software (version 3.5.3, http://www.R-project.org).

## Results

### Identification of HCC Subtypes Based on the Immune Genes

**Figure 1:**
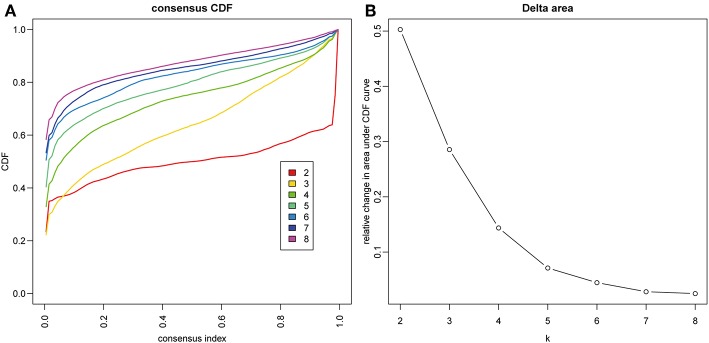
Identification of immune-associated subtypes of HCC in TCGA cohort. **(A)** The cumulative distribution function (CDF) curves is the integral of probability density function, which can completely describe the probability distribution of a real random variable, and established using consensus clustering approach. CDF curves of consensus scores based on different subtype number (*k* = 2, 3, 4, 5, 6, 7, 8) and the corresponding color are represented. **(B)** The CDF Delta area curve of all samples when *k* = 4.

**Figure 2:**
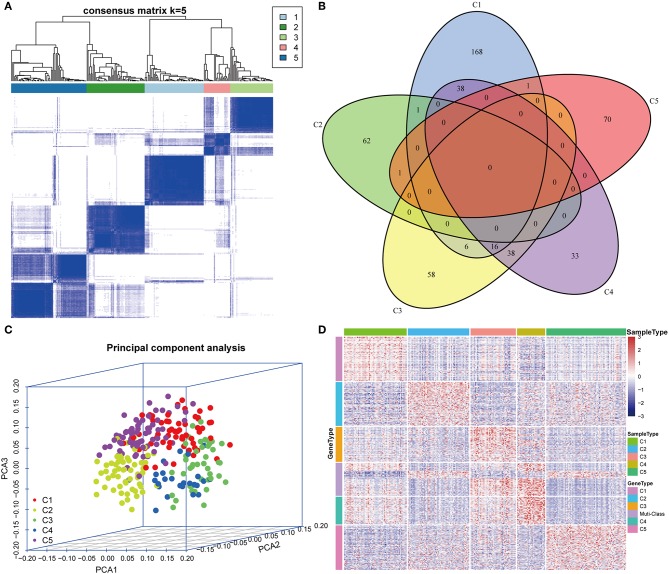
Expression profile analysis of five HCC subtypes. **(A)** The consensus score matrix of HCC samples when *k* = 5 (1 = C1, 2 = C2, 3 = C3, 4 = C4, 5 = C5). A high consensus score between two samples suggests that they have higher probability to be grouped into the same cluster in different iterations. **(B)** Venn diagram of the overlapping significantly upregulated genes in five HCC subtypes. **(C)** Three-dimensional principal component analysis (PCA) of gene expression profile of the upregulated genes. Each sample is represented with a single point, with different color for each of the five subtypes. **(D)** Gene expression heatmap analysis of top 100 genes that were significantly upregulated in each subtype. Heat map indicates relative gene expression value, with red for high expression and blue for low expression.

The gene expression profiles of 778 immune-associated genes were used to investigate the HCC subtypes from the TCGA cohort. All tumor samples were divided into *k* (*k* = 2, 3, 4, 5, 6, 7, 8) different subtypes using Consensus Cluster Plus. The optimal division was reached when *k* = 5 based on the CDF curves of the consensus score (Figure 1A). Moreover, SigClust analysis showed that the consensus clusters (*k* = 5) were significant in all the pairwise comparisons (Figure 1B). There was no significant difference in expression distribution of C1 vs. C5, C4 vs. C5, and C2 vs. C3 (*P* < 0.05). However, significant edge effects were detected in C1 vs. C2, C1 vs. C3, C2 vs. C4, and C3 vs. C5. Thus, the five clusters of samples were separated and the 257 HCC tumor samples extracted from the TCGA cohort were classified into five molecular subtypes underlying the whole immune gene expression profile (Figure 2A).

### Five Subtypes Were Characterized in Immune Microenvironment

The upregulated immune-related genes in each molecular subtype in comparison with other subtypes were analyzed using Kolmogorov–Smirnov test (FDR < 0.05). Among the 778 immune-related genes, 230 genes in subtype C1, 64 genes in subtype C2, 118 genes in subtype C3, 125 genes in subtype C4, and 72 genes in subtype C5 were significantly upregulated. More importantly, 54 genes overlapped between subtypes C3 and C4, and 54 genes overlapped between subtypes C1 and C4. However, only a few overlapped genes were identified in the other pairs of subtypes (Figure 2B). The top 100 upregulated genes from each subtype were selected for three-dimensional PCA and the top two principal components were extracted and visualized using a scatter plot (Figure 2C). PCA results demonstrated that these genes were clearly classified into five subtypes. To further identify the gene expression pattern of each subtype, the selected top 100 genes in each subtype were examined with a heatmap (Figure 2D). The heatmap results showed a distinct expression pattern in the immune upregulated gene profiles of each subtype.

### Clinical Characteristics of the Five Subtypes

**Figure 3:**
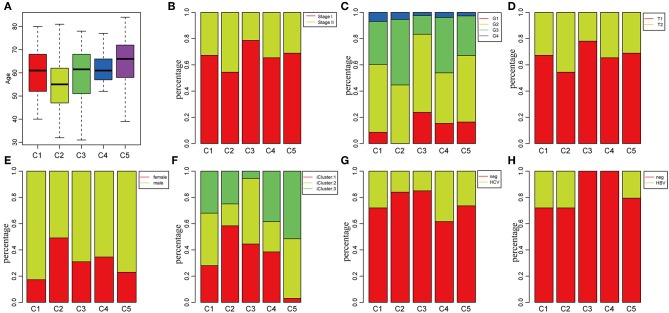
Factor analysis of five HCC subtypes based on clinical characteristics. **(A)** Age distribution in the five HCC subtypes. **(B)** Stage ratio distribution in the five HCC subtypes. **(C)** Histological grade ratio distribution in the five HCC subtypes. **(D)** T stage ratio distribution in the five HCC subtypes. **(E)** Gender ratio distribution in the five HCC subtypes. **(F)** Distribution of subtypes underlying global classification in five subtypes. **(G)** Hepatitis C virus (HCV) distribution in the five HCC subtypes. **(H)** Hepatitis B virus (HBV) distribution in the five HCC subtypes.

To investigate the clinical relevance of tumor microenvironment, clinical factors, including age, gender, tumor, node, metastasis (TNM) staging, and stage in the five subtypes, were analyzed. There was a significant difference in age distribution when the age threshold was set as 60 (chi-square distribution test, *P* = 0.00019) (Figure 3A). The average age in subtype C2 was relatively lower, while it was relatively higher in subtype C5 as compared to other subtypes (Figure 3A). Moreover, the stage relevance in the five subtypes was further analyzed (Figure 3B). The proportion of stage I in subtype C2 and the proportion of stage II in subtype C3 were significantly lower (chi-square distribution test, *P* < 0.001). Furthermore, the grade distribution in the different subtypes was estimated (Figure 3C), and it was revealed that the proportion of G1 in subtype C3 and the proportion of G3 and G4 was significantly higher in comparison with other subtypes (*P* < 0.001).

Of the TNM staging, only one type could not be compared further in the different subtypes in terms of the node and metastasis. The tumor staging in the five subtypes is shown in Figure 3D, and the proportion of T2 in subtype C2 was significantly higher in comparison with the other subtypes (*P* < 0.001). In addition, the relationship between subtypes and gender was analyzed in Figure 3E. The result demonstrated that the proportion of females in subtype C1 and the proportion of males in subtype C2 were significantly higher in comparison with other subtypes (*P* < 0.001). The molecular subtypes of HBV/HCV were further explored for comprehensive genomic analysis of HCCs that were reported in the five subtypes (Figures 3G,H). According to previous studies (35), we classified the five subtypes into three iclusters (Figure 3F). The proportion of icluster1 in subtype C2 and the proportion of icluster3 in subtype C5 were significantly higher in comparison with the other subtypes (*P* < 0.001). However, there was no significant difference in the HBV/HCV proportions among all the subtypes.

### Tumor Immunogenicity of HCC

**Figure 4:**
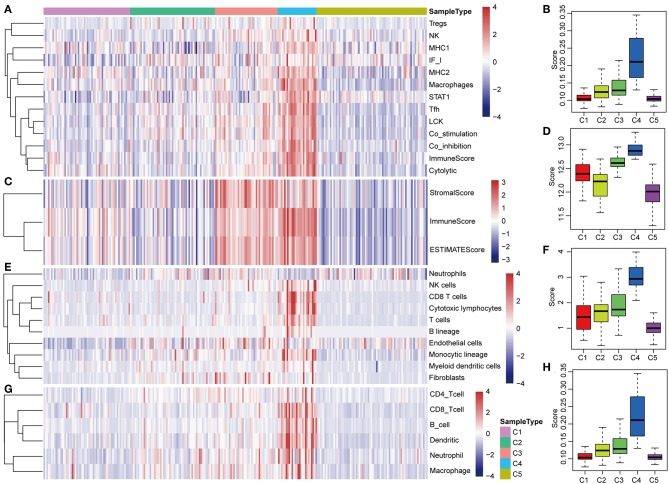
Immune profiles of the five HCC subtypes in the TCGA cohort. **(A)** The gene expression scores of 13 groups of immune metagenes in the five HCC subtypes are displayed in the upper panel. Heatmap indicating the gene expression value, with red reflecting high expression and blue reflecting low expression. From left to right are C1, C2, C3, C4, and C5. **(B)** Boxplot showing the expression scores of 13 groups of metagenes. **(C)** The tumor stroma scores, the immune scores, and the tumor purity of the five HCC subtypes are displayed in the upper panel. **(D)** Boxplots indicating the tumor stroma scores, the immune scores, and the tumor purity of the five HCC subtypes. **(E)** The scores of 10 groups of immune-related cells among the five HCC subtypes are shown in the upper panel. **(F)** Boxplot showing the scores of 10 groups of immune-related cells. **(G)** The scores of six groups of immune cells across five subtypes are shown in the upper panel. **(H)** Boxplots indicating the scores of 10 groups of immune-related cells.

The potential immune escape mechanisms of HCC in the five subtypes were further explored. The scores of 13 types of immune metagenes, tumor immune component (matrix, immunity, tumor purity), 6 types of immune infiltrating cells, and 10 types of immune cell-related MCP counter were collected. Most of the metagenes were overexpressed in subtype C4 and underexpressed in subtype C5 (Figures 4A,B). The comprehensive immune component score was significantly higher in subtype C4 and was significantly lower in subtype C5 compared with other subtypes (Figures 4C,D). Notably, the matrix score in subtype C3 was relatively higher when compared with other subtypes (Figures 4C,D). Of the 10 types of immune cell-related MCP counter, the scores of T cells, and CD8 cells were significantly higher in subtype C4 and were significantly lower in subtype C5 in comparison with the other groups (Figures 4E,F). Of the six types of immune-infiltrating cells, the scores of B cell, CB8 cell, neutrophil, dendritic, and macrophage were significantly higher in subtype C4 and were significantly lower in subtype C5 (Figures 4G,H). In summary, most of the immune signatures were upregulated in subtype C4 and downregulated in subtype C5 in comparison with the other subtypes, which suggested that subtype C4 and C5 had enhanced immune profile and a decreased immune profile (Figure S1).

The expression profiles of eight immune checkpoint genes, which play a key role in immune modulation, were further examined. As shown in **Figure 8A**, PDCD1, CD274, CTLA4, CD86, and CD80 were upregulated significantly in subtype C4 in comparison with the other subtypes, while CD276 was significantly upregulated in subtype C2 in comparison with the other subtypes. Subsequently, the expression values of eight immune checkpoint genes in the five subtypes (**Figure 6B**) were analyzed. There was a significant difference in the expression value of these checkpoint genes except in VTCN1.

### Prognostic Significance of the Five Molecular Subtypes

**Figure 5:**
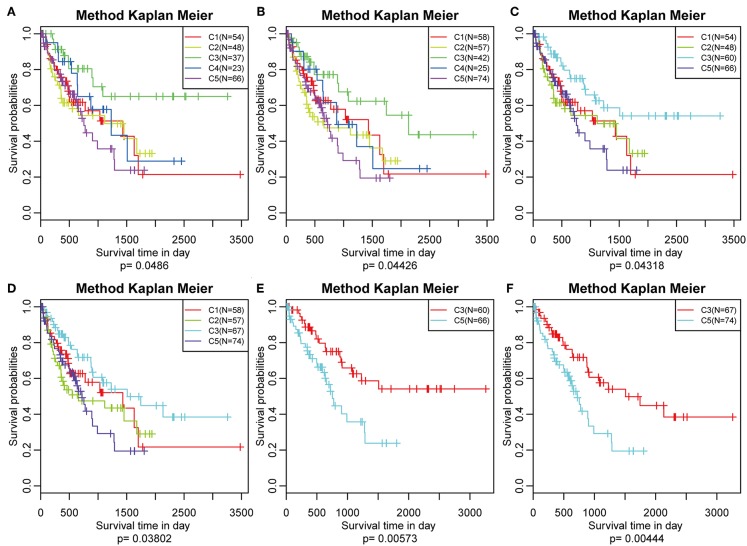
Survival analysis of the five HCC subtypes. Kaplan–Meier curves showing the distinct outcomes of HCC patients in the five molecular subtypes. The *P*-value was calculated using the log-rank test, by comparing the overall five subtypes together or comparing C1, C2, C3, and C5 or subtype C3 vs. subtype C5 only. **(A)** KM curve of disease-free survival (DFS) prognosis of five subtypes. **(B)** KM curve of non-progressive survival prognosis of five subtypes. **(C)** The prognosis difference KM curve of the non-DFS of merged C3, C4, and other subtypes. **(D)** The prognosis difference KM curve of the non-progression-free survival (PFS) of merged C3, C4, and others subtypes. **(E)** The prognosis difference KM curve of the non-DFS of merged C3, C4, and C5. **(F)** The prognosis difference KM curve of the non-PFS of merged C3, C4, and C5.

The poor prognosis in HCC is mainly caused by high incidence of tumor progression and disease recurrence (36). Based on the differently expressed immune profile, the association between clinical outcome of HCC patients and the five subtypes was subsequently investigated. In the TCGA cohort, Kaplan–Meier curves suggested significantly different DFS (log-rank test, DFS, *P* = 0.0486, Figure 5A) and PFS (log–rank test, *P* = 0.04426, Figure 5B) of the HCC patients in the five subtypes. Patients in subtype C5 had the worst outcome, and HCC patients in subtype C3 had the best outcome among all the five subtypes underlying both DFS and PFS. Notably, subtype C4 had the highest immune score but did not show the best outcome, which could have been as a result of the relatively small sample size. The samples in subtype C3 and C4, which had relatively high immune scores, were combined and the clinical outcome of HCC patients in the four groups were further investigated. More significant difference in DFS (log-rank test, *P* = 0.04318) and PFS (log-rank test, *P* = 0.03802) are shown in Figures 5C,D, suggesting that high immune scores in early liver cancer could be a protective factor in early HCC. To explore the relationship between clinical outcome and immune score, DFS and PFS in subtypes C3/C4 with high immune scores and subtype C5 with a low immune score were compared and it was found that subtypes C3/C4, two immune-enhanced molecular subtypes, showed better clinical outcome (log-rank test, DFS, *P* = 0.0573; PFS, *P* = 0.00444, Figures 5E,F).

### Frequencies of Mutant Genes of the Five Molecular Subtypes

**Figure 6:**
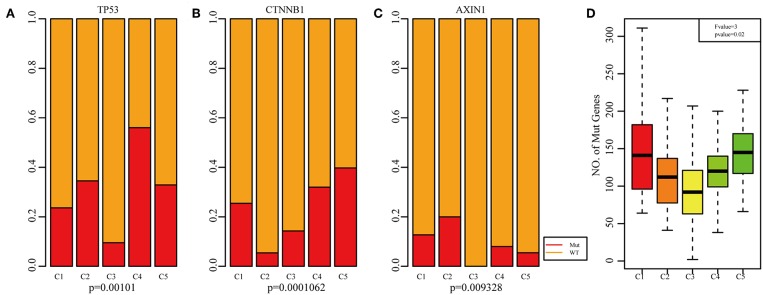
Mutation analysis for the five subtypes of HCC. Red represents the mutation. The mutation ration of TP53 **(A)**, CTNNB1 **(B)**, and AXIN1 **(C)** in the five HCC subtypes. **(D)** Frequency of gene mutation across the five HCC subtypes.

**Figure 7:**
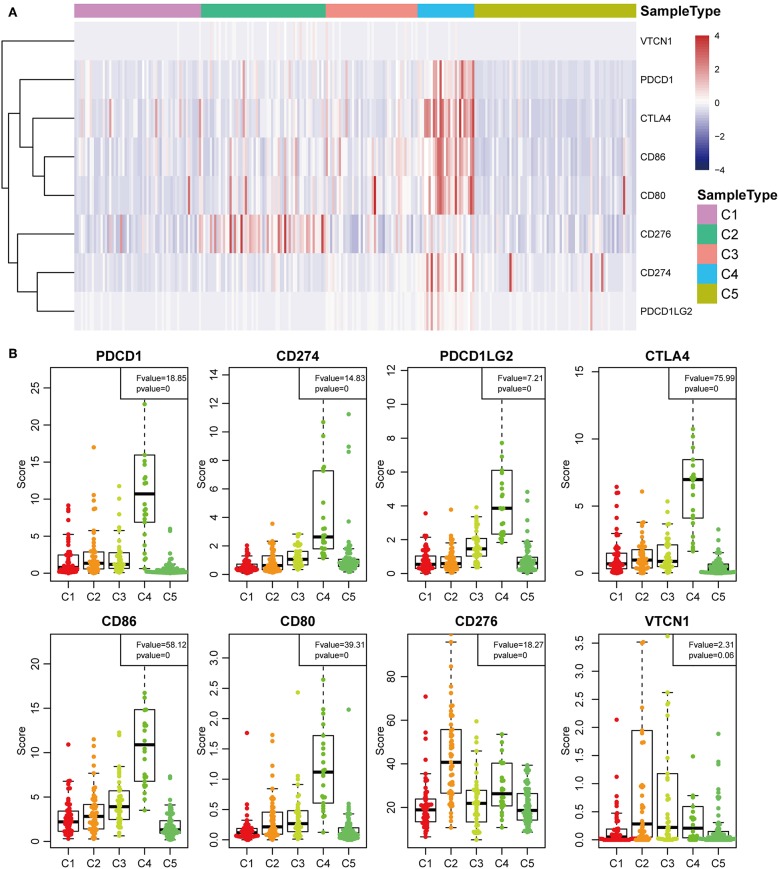
Identification of the five HCC subtypes in THE TCGA cohort. **(A)** Gene expression profile of eight checkpoint molecules (VTCN1, PDCD1, CTLA4, CD66, CD60, CD276, CD274, and PDCD1LG2) across five HCC subtypes in the TCGA cohort. Five subtypes (columns) are presented by the centroids of the TCGA cohort. Heatmap showing the associated gene expression value, with red denoting high expression and blue denoting low expression. **(B)** The expression profile scores of eight checkpoint molecules (PDCD1, CD274, PDCD1LG2, CTLA4, CD86, CD80, CD276, and VTCN1) in the five HCC subtypes.

Previous studies have reported that TP53 (37), CTNNB1 (38), and AXIN1 (39–41) mutations are closely related to the development of HCC. The genomic mutations of these three genes in the five subtypes were subsequently investigated. The results showed that there was significant difference in sample proportions of TP53, CTNNB1, and AXIN1 mutations as well as non-mutations among the five subtypes (Figures 6A–C). The proportion of TP53 mutations in the subtype C3 was significantly lower than that of the other subgroups; the proportion of CTNNB1 mutations in subtypes C2 and C3 was significantly lower than that of the other subtypes; and the proportion of AXIN1 mutations in the subtypes C2 was significantly higher than that of the other subtypes. Notably, there were no mutations in subtype C3. Moreover, there was a significant difference in the frequencies of the mutant genes in the five subtypes (Figure 6D). Similarly, the mutant frequencies in the subtype C3 were significantly lower compared with that of the other subtypes (*P* = 0.02). We further analyzed the relationship between the expression of 8 immune checkpoints in these five subtypes. As shown in Figure 7A, PDCD1, CD274, PDCD1LG2, CTLA4, CD86, and CD80 were significantly higher in C4 than in other types, and CD276 was expressed higher in C2 than in other groups. Further analysis of the gene expression distribution of the eight immune checkpoints is shown in Figure 7B. It can be seen that there is a significant difference in the expression distribution among the five types of samples except VTCN1.

### Gene Co-expression Network Analysis

**Figure 8:**
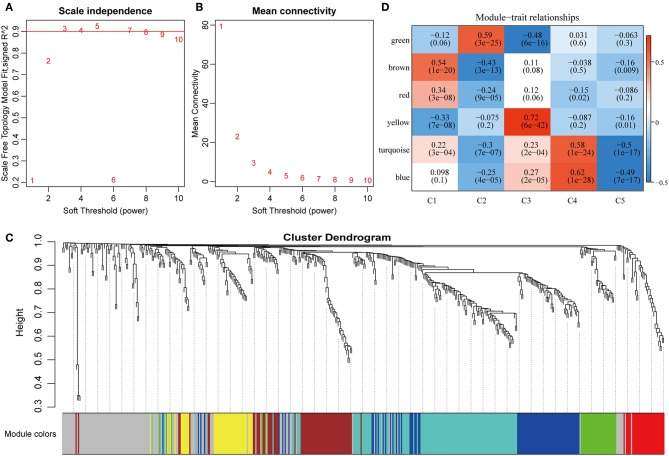
Weighted gene co-expression network analysis (WGCNA) of differentially expressed immune-related genes in the five HCC subtypes in TCGA cohort. Analysis of network topology for various soft thresholding powers **(A,B)**. **(C)** Hierachical cluster tree displaying seven modules of co-expressed genes. The seven modules were validated and are designated by the following colors: “Brown,” “Yellow,” “Blue,” “Red,” “Green,” “Turquoise,” and “Gray.” **(D)** Heatmap showing the correlation between feature vectors of six modules (except gray modules) and five HCC subtypes.

To further explore the potential markers associated with the immune microenvironment of HCC, the data on the expression profiles of a total of 492 immune-related differentially expressed genes were obtained, and the distance between different transcripts was calculated using the Pearson correlation coefficient. The scale-free co-expression network is that logarithm log(*k*) of the node with the connection degree k is negatively correlated with the logarithm log(*P*(*k*)) of the probability of the node, and the correlation coefficient is >0.8. To construct a scale-free network, the value of β was set as 3 (Figures 8A,B). The expression matrix was then converted into an adjacency matrix, and the adjacency matrix was converted into a topological matrix. Based on TOM, the average-linkage hierarchical clustering method was used to cluster the genes according to the criteria of the hybrid dynamic cut tree, and the minimum number of genes was set at 30 per module. After applying the dynamic shear method to determine the gene module, the eigengenes of each module were calculated and the cluster analysis for each module was performed. The closer modules were then merged into new modules, and set height = 0.25, deepSplit = 2, and minModuleSize = 30. Finally, a total of seven modules with all immune-related differentially expressed genes were identified using WGCNA (Figure 8C). Notably, the gray module was a collection of genes that could not be aggregated into other modules. The transcript statistics of each module are shown in Table 1. A total of 371 transcripts were divided into five co-expression modules. The correlation between the eigengenes of six modules and five subtypes (Figure 8D) was calculated. The blue module was positively correlated with subtypes C3 and C4, and was negatively correlated with subtype C5; the yellow module was positively correlated with subtype C3, and was negatively correlated with subtype C5; the turquoise module was positively correlated with subtypes C1, C3, and C4, and was negatively correlated with subtype C5; the green module was positively correlated with subtype C2, and was negatively correlated with subtype C3; the brown and red modules were positively correlated with subtype C1, and was negatively correlated with subtype C2.

**Table 1:** Transcript data for six modules.

**Modules**	**Genes**
Blue	77
Brown	66
Green	34
Red	31
Turquoise	121
Yellow	42

To investigate the biological functions of the five modules, KEGG enrichment analysis was performed. The brown module was mainly enriched in the B cell receptor signaling pathway; the yellow module was mainly enriched in eight pathways closely associated with cancer, such as EGFR tyrosine kinase inhibitor resistance, small cell lung cancer, focal adhesion, and other pathways; the blue module was mainly enriched in 24 pathways, including inflammation-related pathways, such as *Staphylococcus aureus* infection, and intestinal immune network for IgA production; the red module was mainly enriched in cell adhesion molecules (CAMs), transcriptional misregulation in cancer, and inflammatory mediator regulation of TRP channels; while the turquoise module was mainly enriched in 21 pathways, such as T cell receptor signaling pathway, CAMs, Th1, and Th2 cell differentiation, cytokine–cytokine receptor interaction and other immune-related pathways.

**Figure 9:**
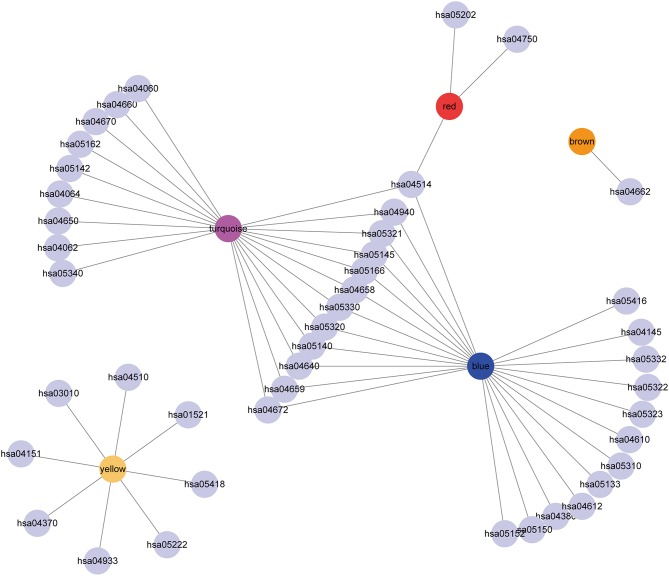
Cytoscape representation of the enriched pathways associated with co-expressed genes in the seven modules.

Subsequently, the relationship network of enriched pathways in these modules was visualized. As shown in Figure 9, it was found that these modules were enriched in 44 pathways. There were 12 common pathways enriched in blue and turquoise modules. Few intersections of the enriched pathways were enriched in other modules, suggesting that the genes in the blue and turquoise modules may share similar regulatory processes in the five subtypes.

### External Validation of the Five Subtypes

A total of 73 featured genes with a correlation coefficient >0.8 were obtained from the co-expression gene modules (blue, brown). The expression profile of the featured genes was further extracted to serve as a training set and employed SVM to classify the samples. The classification accuracy rate of SVM was 91.1%. To further validate the five subtypes, 170 samples were classified using SVM. Thirty-nine samples were predicted in subtype C1, 40 samples were predicted in subtype C2, 18 samples were predicted in subtype C3, 29 samples were predicted in subtype C4, and 44 samples were predicted in subtype C5.

**Figure 10:**
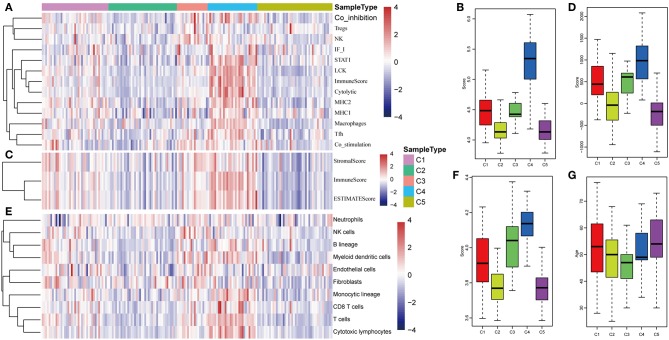
Immune profiles of five HCC subtypes in the validation set. **(A)** The gene expression scores of 13 groups of immune metagenes in the five HCC subtypes are displayed in the upper panel. Heatmap showing the gene expression value, with red denoting high expression and blue denoting low expression. **(B)** Boxplot showing the expression scores of 13 groups of metagenes. **(C)** The tumor stroma scores, the immune scores, and the tumor purity in the five HCC subtypes are displayed in the upper panel. **(D)** Boxplots indicating the tumor stroma scores, the immune scores, and the tumor purity of the five HCC subtypes. **(E)** The scores of 10 groups of immune-related cells across the five HCC subtypes are shown in the upper panel. **(F)** Boxplot shows the scores of 10 groups of immune-related cells. **(G)** The HCC sample age distribution of five subtypes in the validation set.

**Figure 11:**
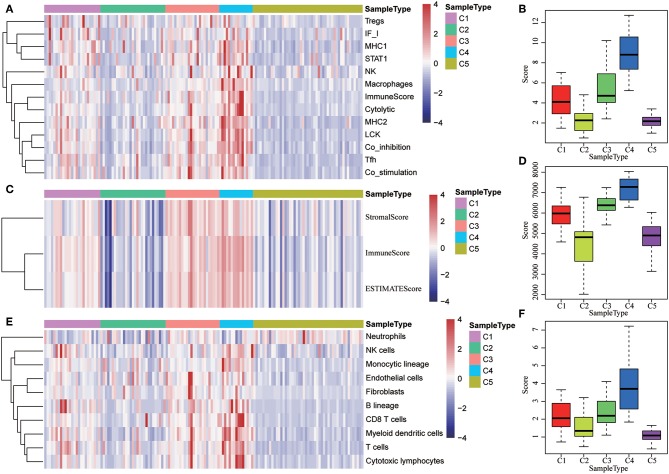
The expression profiles of immune metagenes in the five HCC subtypes in validation set from ICGC. **(A)** The expression of 13 types of immune metagenes in five subtypes and the median value of the 13 categories of immunoreagents in the sample of the five subtypes in the validation set. **(B)** The median value of the 13 categories of immunoreagents in the sample of the five subtypes in the validation set. **(C)** The matrix scores, immune scores, and tumor purity score in five subtypes in validation set. **(D)** The matrix scores, immune scores, and median values of tumor purity scores in five subtypes in the validation set. **(E)** The scores of 10 types of immune-related cells in five subtypes in validation set. **(F)** The distribution of the median value of the 10 types of immune-related cells in the sample of the five subtypes in validation set.

The expression distribution of 13 immune metagenes in the five subtypes was subsequently analyzed as was shown in Figures 10A–D. Most of the metagenes were highly expressed in subtype C4, which was consistent with the result in training set. Moreover, the immune scores were investigated as was shown in Figure 10B. It was found that the immune score in subtype C4 group was significantly higher as compared with the other subtypes, and the matrix score of subtype C3 was significantly higher in comparison with the other subtypes, which was also consistent with the training set. Further analysis of the sample immune scores is shown in Figures 10C,D; the results showed that the immune score of subtype C4 was significantly higher in comparison with the other subtypes, and the matrix score of subtype C3 was significantly higher than that of the other subtypes, which was consistent with the training set. Moreover, the distribution of 10 immune-related cells in the five subtypes of samples was analyzed as shown in Figures 10E,F. Similar with the training set, it was found that the proportion of most of the cells was higher in subtype C4 as compared with the other subtypes. Finally, the age distribution of the five subtypes was analyzed and the post-distribution difference is shown in Figure 10G. It was found that the age distribution of the five subtypes was also consistent with that of the training set. Similar with these results, the expression profiles of immune metagenes in the five HCC subtypes were further validated in the ICGC database in Figures 11A–F. These data suggested that there were immune-enhanced subtypes and immune-decreased subtypes in early HCC, and there was a significant difference between the two subtypes.

## Discussion

HCC is an aggressive malignancy, which is still the third leading cause of tumor-related deaths. Although there have been advances in treatment strategies, no effective molecular targeted therapy has been successfully validated. The intricate microenvironment, sustained by the production of growth factors with parenchyma, as well as the infection of hepatitis viruses, promotes the occurrence and development of HCC (42). The current study aimed at systematically analyzing the heterogeneous HCC microenvironment subtypes underlying global immune genes in stage I/II and related clinical significance using multi-omics data extracted from the TCGA cohort. Five molecular subtypes in immune microenvironment were found to exhibit significantly different clinical characteristics, immune escape mechanisms, genomic alterations, signaling, and outcomes. Subtype C4 was found to be an immune-enhanced subtype while subtype C5 was found to be an immune-decreased subtype in the immune microenvironment of HCC. The TP53, CTNNB1, and AXIN1 mutations were closely associated with immune-enhanced molecular subtypes. Finally, these five subtypes were validated in an external cohort of NCI.

Many previous studies have revealed some prognostic subtypes and histological subtypes of HCCs underlying the genome-wide profiles (43–45). The current study mainly aimed to investigate the global immune profiles for a more comprehensive analysis of immune landscape in HCC. A total of five molecular subtypes were identified. The results revealed that the immune profile of subtype C4 was significantly higher in comparison with that of the other molecular subtypes, and the immune profile of subtype C5 was significantly lower than that of the other subtypes. Moreover, subtype C4 showed increased immune cell infiltration score (including tumor matrix, immunity, purity), which was positively related to the expression signatures of multiple types of immune-related cells. These results demonstrated that subtype C2 was correlated with an enhanced immune status in the HCC immune microenvironment. On the contrary, subtype C5 showed lower expression of immune profiles and had the lowest immune cell infiltration score among the five subtypes, which was negatively related to expression signatures of the selected immune-related cells. Consistent with the findings of the training set, the validation set also revealed that most metagenes were highly expressed and showed higher immune scores in subtype C4 than that of the other four subtypes. Thus, it was hypothesized that both immune-enhanced subtype and immune-decreased subtype existed in the HCC immune microenvironment and were significantly different in terms of expression profile of metagenes, the immune components score, the immune infiltration score, and the MCP counter of immune cells.

This study further investigated the potential immune escape molecular mechanisms of HCC. It is well-known that there are two main aspects of intrinsic immune escape, including immunogenicity and the expression of immune checkpoint molecules (46). Mutations of TP53, CTNNB1, and AXIN1 gene in HCC were validated by whole genome, exome, and transcriptome sequencing (40, 41, 47). However, the roles played by these gene mutations in the molecular mechanism of immune microenvironment of HCC have not been studied to date. This study revealed that the five molecular subtypes were significantly different in terms of the mutations and frequencies of TP53, CTNNB1, and ACIN1. Previous studies reported that immune checkpoints are often activated in HCC with high immune response accompanied with upregulated gene expression (48). The most common immune checkpoint receptors are BTLA, VISTA, PD-1, CTLA-4, LAG-3, TIM-3, and OX40. The current study revealed significant overexpression of checkpoint genes, including PDCD1, CD274, PDCD1LG2, CTLA4, CD86, and CD80 in subtype C4 than in the other subtypes. These results suggest that the immune-enhanced subtype C4 may be closely associated with intrinsic immune escape of HCC, which may provide new insights into immunotherapy of HCC using immune checkpoint blockers.

Valerie and colleagues reported that the survival outcome of HCC patients is positively correlated with higher expression of a group of innate immune-related and inflammatory genes, including NK-associated molecules and macrophage (13). Moreover, many other targets have been considered as prognostic indicators for HCC, including immunoregulatory enzyme indoleamine 2,3-dioxygenase (IDO) (49), epithelial neutrophil-activating peptide-78 (CXCL5) (50), CXCR6 (51), and so on. It was found in the current study that these five molecular subtypes were consistently associated with different survival outcomes in HCC patients. Subtype C5 had the worst survival outcome while subtype C3 had the best outcome among all the five subtypes. Notably, subtypes C3 and C4, which had higher immune scores, showed better survival outcome compared with subtype C5, which suggests that the subtype with high immune score may play a protective role in the early stage of HCC. Thus, this study may provide immune signature for survival prediction in the early stage of HCC.

The current research also explored the potential targets and pathways of the five subtypes in the HCC immune microenvironment using WGCNA analysis. The immune-related genes were enriched in six different modules, and the results revealed that inflammatory pathways were mainly enriched in the blue module, and the immune-related pathways were mostly enriched in the turquoise module. The blue and turquoise modules shared the most common pathways of all pairwise comparisons, which suggests that the genes in these two modules may play similar roles in the HCC immune microenvironment. Consistent with the current results, previous studies reported that the tumor immune microenvironment plays key roles in regulating the process of hepatocarcinogenesis, tumor invasion, and metastasis (52). Innate immunity plays a critical role in modulating HCC tumor occurrence and development because the liver is an organ with predominant innate immunity (53). It has also been hypothesized that the adaptive immune response may be induced in the progression of HCC (54). Therefore, clarifying the molecular mechanisms based on the immune microenvironment of HCC may facilitate identification novel therapeutic and chemopreventive targets for HCC.

There are some limitations in the current study. Firstly, to comprehensively reflect the factors and effects influencing HCC microenvironment phenotypes, more clinical characteristics and demographic features of HCC patients should be included in subgroup analysis. Secondly, the sample size in each subtype was relatively small in training as well as validation set, and only the NCI and ICGC cohorts were used for external validation, which may have resulted in one-sided results and a high false-positive rate. It will be helpful to perform cross-validation in internal validation and increase the sample size for external validation in future studies on the immune microenvironment of HCC. Finally, more experimental evidence for immunogenomic analysis is needed to validate the roles of mutation genes, checkpoint genes, and the enriched pathways involved in immune microenvironment.

## Conclusion

The current study suggests that microenvironment phenotypes of HCC could be classified into five molecular subtypes with potential immune escape mechanisms in HCC. These subtypes are distinct in immunity characteristics, immune checkpoint molecules, and patient outcomes. Moreover, specific functional pathways may drive the formation of microenvironment phenotypes. These results may provide guidance for developing novel strategies of immunotherapy in HCC.

## Data Availability Statement

Publicly available datasets were analyzed in this study. This data can be found here: TCGA-LIHC, GSE14520, and ICGC-LIRI-JP.

## Author Contributions

JL and WL designed experiments and interpreted data. JL, HW, and ZM conducted bioinformatic and statistical analyses. HW, JZ, WO, and YQ wrote the paper. All authors have read and approved the manuscript for publication.

### Conflict of Interest

The authors declare that the research was conducted in the absence of any commercial or financial relationships that could be construed as a potential conflict of interest.

## Supplementary Material

Figure S1 Boxplots indicating the infiltrating immune scores of all types of immune metagenes among the five subtypes.

Table S1 Patients' information in the TCGA, GSE14520, and ICGC cohorts.
