# Supplementary figures and images for: Multi-omics Analysis of Microenvironment Characteristics and Immune Escape Mechanisms of Hepatocellular Carcinoma
Source: Front Oncol. 2019 Oct 15;9:1019. doi: 10.3389/fonc.2019.01019 (PMC6803502; doi:10.3389/fonc.2019.01019)

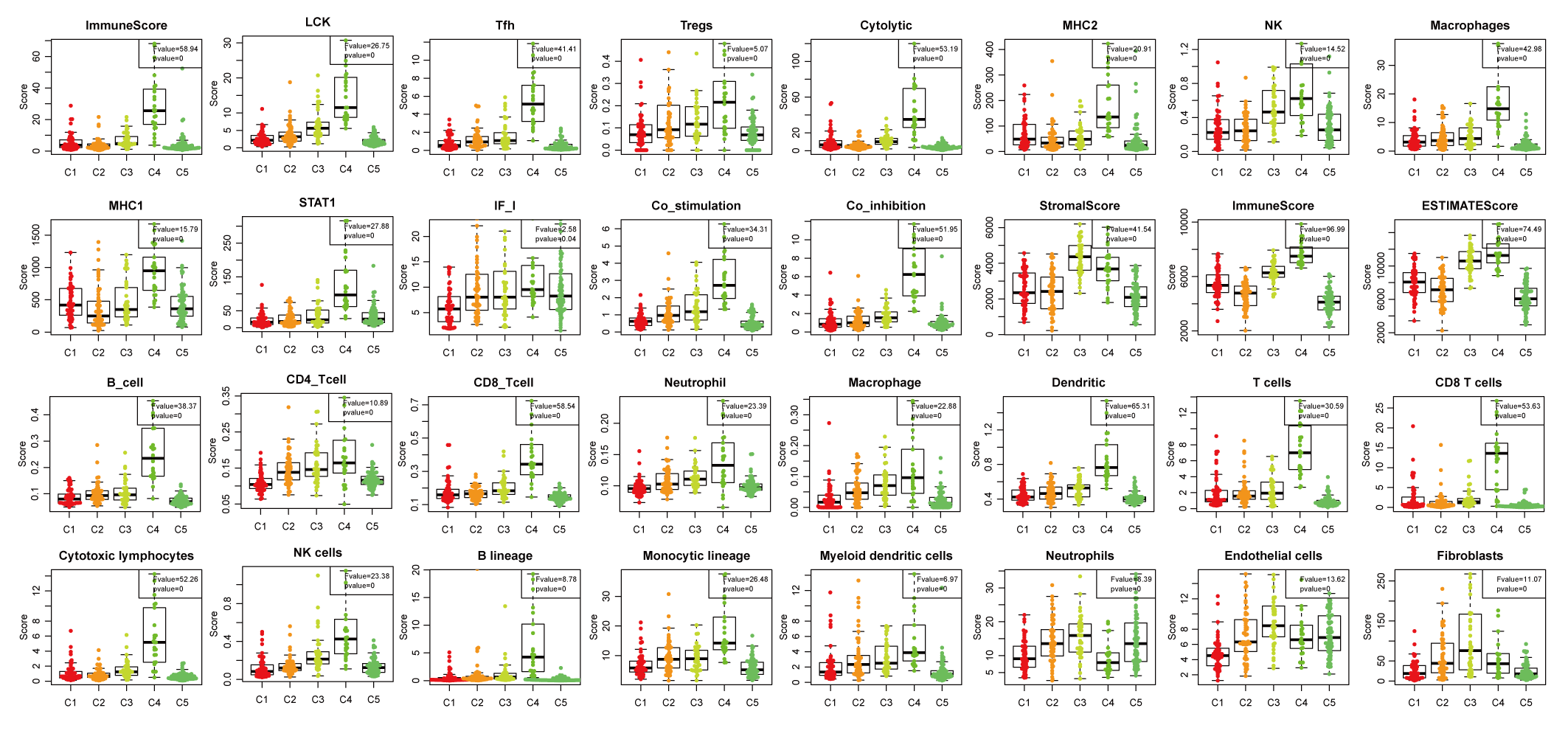

Supplement: Figure S1 — Boxplots indicating the infiltrating immune scores of all types of immune metagenes among the five subtypes. [file Image_1.TIF]
