# Supplementary material for: Multi-omics Analysis of Microenvironment Characteristics and Immune Escape Mechanisms of Hepatocellular Carcinoma
Source: Front Oncol. 2019 Oct 15;9:1019. doi: 10.3389/fonc.2019.01019 (PMC6803502; doi:10.3389/fonc.2019.01019)
Supplement: Table S1 — Patients' information in the TCGA, GSE14520, and ICGC cohorts. [file Table_1.DOCX]

| Clinical characteristics |  | Total | % |
| --- | --- | --- | --- |
| TCGA |  | 257 |  |
| Age | <=65 years | 160 | 62.26 |
|  | >65 years | 97 | 37.74 |
| Gender | Male | 180 | 70.04 |
|  | Female | 77 | 29.96 |
| Histological grade | G1 | 31 | 12.06 |
|  | G2 | 127 | 49.42 |
|  | G3 | 86 | 33.46 |
|  | G4 | 11 | 4.28 |
| Stage | Ⅰ | 171 | 66.54 |
|  | Ⅱ | 86 | 33.46 |
| T classification | T1 | 170 | 66.15 |
|  | T2 | 86 | 33.46 |
| HCV | Neg | 89 | 34.63 |
|  | Pos | 28 | 10.89 |
| HBV | Neg | 96 | 37.35 |
|  | Pos | 21 | 8.17 |
| GSE14520 |  | 170 |  |
| Stage | Ⅰ | 93 | 54.70 |
|  | Ⅱ | 77 | 45.30 |
| Gender | Female | 27 | 15.88 |
|  | Male | 143 | 84.12 |
| Age | <=65 | 151 | 88.82 |
|  | >65 | 19 | 11.18 |
| ICGC |  | 142 |  |
| Stage | Ⅰ | 36 | 25.35 |
|  | Ⅱ | 106 | 74.65 |
| Gender | Female | 45 | 31.69 |
|  | Male | 97 | 68.31 |
| Age | <=65 | 51 | 35.92 |
|  | >65 | 91 | 64.08 |

**Table S1** Patients’ information in the TCGA,GSE14520 and ICGC cohorts.
